# Hydrogen Sulfide Improves the Cold Stress Resistance through the CsARF5-CsDREB3 Module in Cucumber

**DOI:** 10.3390/ijms222413229

**Published:** 2021-12-08

**Authors:** Xiaowei Zhang, Xin Fu, Fengjiao Liu, Yanan Wang, Huangai Bi, Xizhen Ai

**Affiliations:** State Key Laboratory of Crop Biology, Key Laboratory of Crop Biology and Genetic Improvement of Horticultural Crops in Huanghuai Region, College of Horticulture Science and Engineering, Shandong Agricultural University, Tai’an 271018, China; 2019010077@sdau.edu.cn (X.Z.); 15621321275@163.com (X.F.); lfjsdnd@126.com (F.L.); 18864805562@163.com (Y.W.); bhg163@163.com (H.B.)

**Keywords:** ARF, auxin, cold stress, cucumber, DREB, hydrogen sulfide, module, resistance

## Abstract

As an important gas signaling molecule, hydrogen sulfide (H_2_S) plays a crucial role in regulating cold tolerance. H_2_S cooperates with phytohormones such as abscisic acid, ethylene, and salicylic acid to regulate the plant stress response. However, the synergistic regulation of H_2_S and auxin in the plant response to cold stress has not been reported. This study showed that sodium hydrosulfide (NaHS, an H_2_S donor) treatment enhanced the cold stress tolerance of cucumber seedlings and increased the level of auxin. *CsARF5*, a cucumber auxin response factor (ARF) gene, was isolated, and its role in regulating H_2_S-mediated cold stress tolerance was described. Transgenic cucumber leaves overexpressing *CsARF5* were obtained. Physiological analysis indicated that overexpression of *CsARF5* enhanced the cold stress tolerance of cucumber and the regulation of the cold stress response by *CsARF5* depends on H_2_S. In addition, molecular assays showed that *CsARF5* modulated cold stress response by directly activating the expression of the dehydration-responsive element-binding (DREB)/C-repeat binding factor (CBF) gene *CsDREB3*, which was identified as a positive regulator of cold stress. Taken together, the above results suggest that CsARF5 plays an important role in H_2_S-mediated cold stress in cucumber. These results shed light on the molecular mechanism by which H_2_S regulates cold stress response by mediating auxin signaling; this will provide insights for further studies on the molecular mechanism by which H_2_S regulates cold stress. The aim of this study was to explore the molecular mechanism of H_2_S regulating cold tolerance of cucumber seedlings and provide a theoretical basis for the further study of cucumber cultivation and environmental adaptability technology in winter.

## 1. Introduction

Cucumber (*Cucumis sativus* L.) is one of the most important economic crops worldwide. The cultivation and yield of cucumbers in China have ranked among the top in the world for many years. Cucumbers are typical cold-sensitive plants and are generally grown in solar greenhouses in northern China. Because of the extreme low-temperature conditions, cucumbers in greenhouses are prone to cold injury in winter and early spring. Cucumbers with cold injury showed inhibited growth, wilted and died in severe cases. Therefore, it is of great practical significance to study the effects of low-temperature stress on cucumber growth and development and the response mechanism of cucumber to low-temperature stress.

Hydrogen sulfide (H_2_S) is a gaseous compound recognized as the third gas signaling molecule discovered after nitric oxide and carbon monoxide [1,2]. H_2_S has been found to be widespread in mammals and has important cellular protective effects [3,4]. However, H_2_S is also highly toxic. Low concentrations of H_2_S can affect the eyes, respiratory system and central nervous system. Inhaling hydrogen sulfide in small concentrations can be fatal [5,6]. In agricultural production, the application of an appropriate concentration of H_2_S can regulate plant growth and development, such as germination, maturation, root development, senescence and defense [7,8,9]. Numerous investigations have determined that H_2_S plays a key role in the regulation of abiotic stress responses, including cold tolerance [8,10,11,12]. Low temperatures trigger H_2_S biosynthesis [13,14]. H_2_S is found to alleviate cold stress tolerance in many plant species, although the mechanisms remain elusive [11,13,14,15]. Several reports show that H_2_S modulates cold stress response, possibly through mitogen-activated protein kinase (MAPK) signaling [11,16,17]. In addition to H_2_S, phytohormones, especially auxin, also play a vital positive regulatory role in cold stress response [18,19]. 

Auxin is involved in plant growth and development in various aspects, including cell division and elongation, tissue patterning and the response to environmental stimuli [20,21]. Since auxin was identified for the first time in the 1930s as indole-3-acetic acid, there has been a major breakthrough in the molecular mechanisms of auxin perception and signal transduction. Many genetic and biochemical approaches have elucidated that the TRANSPORT INHIBTOR RESPONSE1 (TIR1) protein functions as the receptor to perceive auxin signaling based on the reduced auxin response of *tir1* mutations [22,23]. Additionally, the SCF^TIR1^ ubiquitin-ligase complex is regarded as a central regulator of auxin signaling, and it-mediated proteolysis of auxin/indole acetic acid (Aux/IAA) proteins is responsible for auxin signaling transduction [24,25]. In this signaling pathway, Aux/IAA proteins are direct targets of TIR1. Aux/IAA proteins directly interact with auxin response factors (ARFs) to repress their activities [26]. Upon exposure to auxin, the F-box protein TIR1 recruits the Aux/IAA proteins for degradation, which leads to the release of various auxin response factors, including *Small Auxin-up RNAs* (*SAURs*), *GH3s* and *Aux/IAAs*, and consequently regulates diverse auxin-mediated plant growth [27,28].

ARFs are vital transcription factors (TFs) that regulate the expression of auxin response genes [27,29,30]. To date, 23 and 25 *ARF* genes have been isolated in *Arabidopsis* and rice, respectively [27,31,32]. Most ARF members consist of a DNA-binding domain (DBD), a variable middle region and a carboxy-terminal dimerization domain (CTD) [27,33]. The DBD is classified as a plant-specific B3-type and functions to bind to TGTCTC/GAGACA sites (AuxREs) in vitro [33,34]. The middle region includes two types: activation domain-type (AD) and repression domain-type (RD), which are used as the basis of classification between transcription activators and transcription repressors [30,33]. Additionally, the CTD domain is responsible for protein-protein interactions by dimerizing with Aux/IAA proteins as well as other ARFs [35,36]. Extensive studies have suggested that ARF proteins are involved in distinct developmental processes. In *Arabidopsis*, numerous ARF genes have been implicated in embryogenesis (ARF5 and ARF17) [37], root growth (ARF7, ARF10, ARF16, and ARF19) [38,39,40,41], hypocotyl growth (ARF6, ARF7, ARF8, and ARF19) [42,43,44], shoot regeneration (ARF4 and ARF5) [45], flower development (ARF2, ARF3, ARF6, and ARF8) [46,47] and senescence (ARF1 and ARF2) [48]. In the case of rice, genetic studies show that the functions of ARFs are different from the functions of *Arabidopsis*. OsARF1 is involved in root initiation and seed development [49,50]. OsARF12 regulates root elongation, iron accumulation and phosphate homeostasis [51,52]. OsARF16 regulates phosphate transport, phosphate starvation and iron deficiency responses [53,54,55]. OsARF19 controls leaf angles [56]. OsARF11, OsARF12, OsARF16 and OsARF17 are involved in antiviral defences [57,58]. A recent study showed that OsARF6 regulates rice yields [59]. Although a number of ARF members have been functionally characterized in *Arabidopsis* and rice, as mentioned earlier, little is known about the functions of *ARF* genes in cucumber.

In this study, the molecular mechanisms by which H_2_S regulates cold stress response in cucumber were explored. The study suggested that H_2_S treatment could improve cold resistance and auxin content of cucumber. *CsARF5*, a transcriptional regulator in auxin signaling, was responsive to cold stress and H_2_S treatments, and overexpression of *CsARF5* improved the cold stress tolerance of cucumber. Further studies indicated that *CsARF5* modulated cold stress response by directly activating the expression of the dehydration-responsive element-binding (DREB)/C-repeat binding factor (CBF) gene *CsDREB3*.

## 2. Results

### 2.1. Sodium Hydrosulfide (NaHS) Improves Cold Tolerance in Cucumber Seedlings

**Figure 1:**
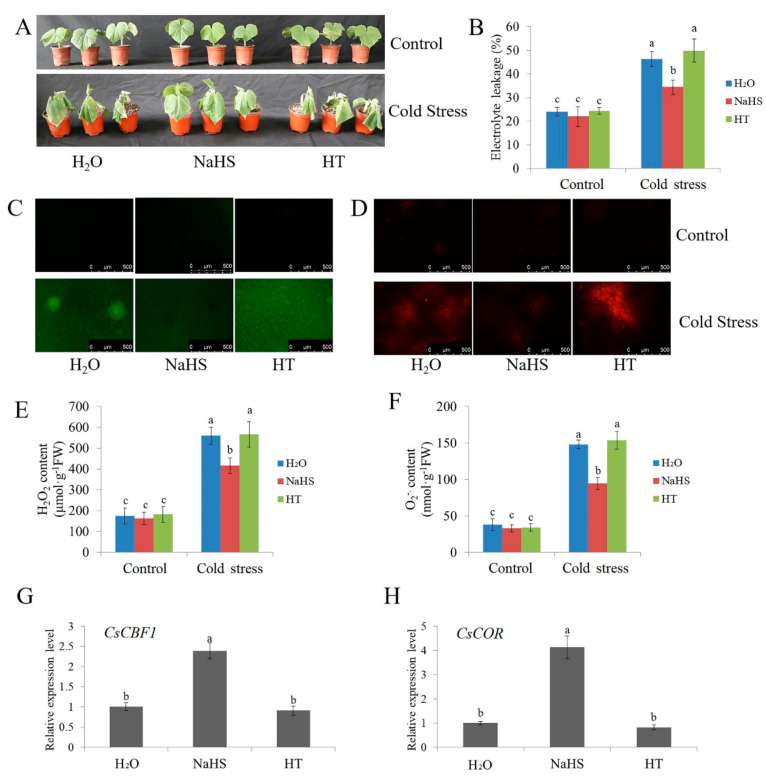
Effects of NaHS and HT on the cold tolerance of cucumber seedlings. Cucumber seedlings were treated with NaHS, HT or deionized water for 24 h and subsequently were exposed to cold stress. (**A**) Phenotypic characterization of cucumber seedlings before (control) and after cold stress (5 °C for 48 h). Each treatment contained 5–10 cucumber seedlings. The experiments were repeated three times with similar results. A typical picture is shown here. (**B**) The EL results of cucumber seedlings before (control) and after cold stress for 48 h. (**C**,**D**) Inverted fluorescence microscopy imaging of H_2_O_2_ and O_2_^·−^ levels in cucumber seedling leaves before (control) and after cold stress treatment for 48 h. (**E**,**F**) Detection of H_2_O_2_ and O_2_^·−^ content of cucumber seedlings before (control) and after cold stress treatment for 48 h. (**G**,**H**) Expression of *CsCBF1* and *CsCOR* genes in cucumber seedlings under cold stress treatment for 6 h. qRT-PCR was performed simultaneously with three biological replicates and three technical replicates. The value of the water treatment was used as the reference and was set to 1. Error bars denote standard deviations. Different letters indicate significant differences (*p* < 0.05) based on Duncan’s multiple range tests.

A previous study demonstrated that NaHS could improve the cold tolerance of cucumber seedlings in a concentration-dependent manner, and 1.0 mM NaHS treatment showed a very significant difference compared with the control [60]. Here, Figure 1 shows that 1.0 mM NaHS significantly reduced cold stress injury, accumulation of H_2_O_2_ and superoxide anion (O_2_^.−^), as well as electrolyte leakage (EL) in cucumber seedlings after exposure to 5 °C for 48 h. However, 0.15 mM H_2_S scavenger hypotaurine (HT) increased cold stress injury, H_2_O_2_, O_2_^.−^ and EL, compared with the deionized water (H_2_O, as a comparison)-treated seedlings (Figure 1A–F). The mRNA abundance of *CsCBF1* and *CsCOR* in NaHS-treated seedlings also increased by 1.38-fold and 3.14-fold, respectively, under cold stress, but no obvious differences were observed between H_2_O and HT treatments (Figure 1G,H). Therefore, the results further confirmed that H_2_S improves cold tolerance in cucumber.

### 2.2. NaHS Treatment Affects Auxin Signaling

**Figure 2:**
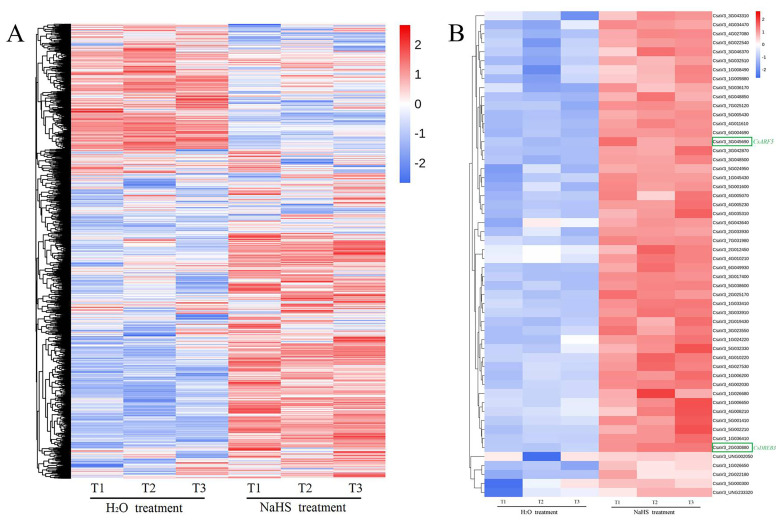
RNA-seq analysis of cucumber seedlings with or without NaHS treatment. (**A**) Normalized heat map showing the changes in gene expression after NaHS treatment at 5 °C for 6 h. All experiments were performed in triplicate. (**B**) Normalized heat map of upregulated gene expression after NaHS treatment at 5 °C for 6 h. All experiments were performed in triplicate.

To explore the molecular mechanism by which H_2_S improves cold resistance in cucumber, transcriptome analyses of cucumber seedlings treated with NaHS and H_2_O were performed. A total of 1952 cucumber genes were analyzed from transcriptome data (Figure 2A). Among these cucumber genes, 118 genes were downregulated, and 54 genes were upregulated (Figure 2A). The upregulated genes were further analyzed (Figure 2B). One of the genes that caught our attention was an auxin response gene (accession number: CsaV3_3G045690, Figure 2B). The NCBI database comparison found that it was the *CsARF5* gene.

**Figure 3:**
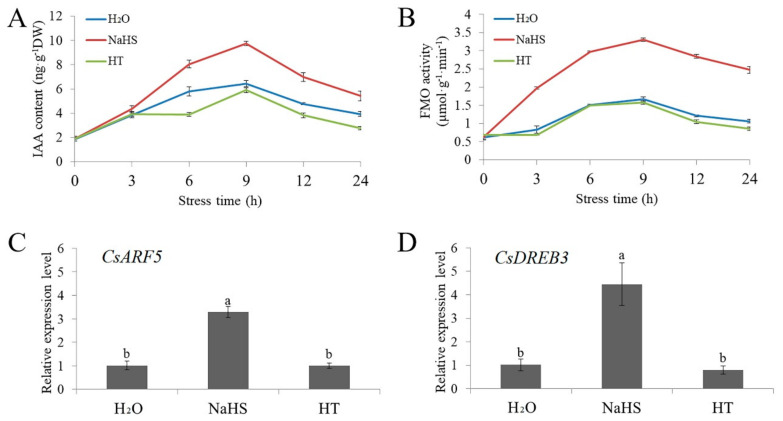
NaHS treatment affects IAA levels in cucumber. (**A**) The IAA content of cucumber seedlings treated with NaHS or HT under cold stress treatment for 24 h. (**B**) The FMO activity of cucumber seedlings treated with NaHS or HT under cold stress treatment for 24 h. (**C**) Expression of the *CsARF5* gene in cucumber seedlings treated with NaHS or HT under cold stress treatment for 6 h. (**D**) Expression of the *CsDREB3* gene in cucumber seedlings treated with NaHS or HT under cold stress treatment for 6 h. qRT- PCR was performed simultaneously with three biological replicates and three technical replicates. The value of 0 h was used as the reference and was set to 1. Error bars denote standard deviations. Different letters indicate significant differences (*p* < 0.05) based on Duncan’s multiple range tests.

To study the effect of NaHS on auxin signaling, the change in auxin content in cucumber seedlings treated with NaHS or HT was estimated. NaHS (1.0 mM) markedly increased endogenous indole-3-acetic acid (IAA) accumulation and flavin monooxygenase (FMO, a key enzyme in auxin synthesis) activity. However, HT treatment revealed lower or similar IAA content and FMO activity compared with H_2_O treatment (Figure 3A,B). In addition, NaHS treatment also significantly upregulated the mRNA levels of *CsARF5* and *CsDREB3*, while HT-treated seedlings showed no marked differences in the relative mRNA expression of *CsARF5* and *CsDREB3* relative to the H_2_O treatment (Figure 3C,D). These data indicate that H_2_S affects auxin signaling in cucumber seedlings under cold stress.

### 2.3. IAA Treatment Improves Cold Resistance of Cucumber Seedlings

**Figure 4:**
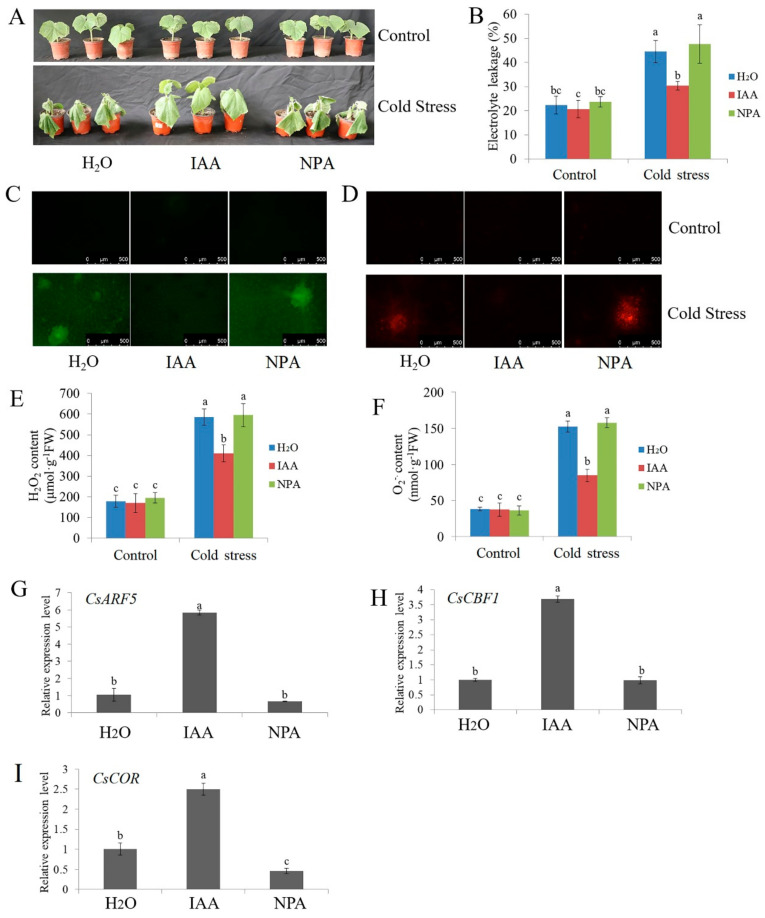
Effects of IAA and NPA on the cold resistance of cucumber seedlings. Cucumber seedlings were treated with IAA, NPA or deionized water for 24 h and subsequently were exposed to cold stress. (**A**) Phenotypic characterization of cucumber seedlings before (control) and after cold stress (5 °C for 48 h). Each treatment contained 5–10 cucumber seedlings. The experiments were repeated three times with similar results. A typical picture is shown here. (**B**) The EL results of cucumber seedlings before (control) and after cold stress treatment for 48 h. (**C**,**D**) Inverted fluorescence microscopy imaging of H_2_O_2_ and O_2_^·−^ levels in cucumber seedling leaves before (control) and after cold stress treatment for 48 h. (**E**,**F**) Detection of H_2_O_2_ and O_2_^·−^ accumulation of cucumber seedlings before (control) and after cold stress treatment for 48 h. (**G**–**I**) Expression of *CsARF5*, *CsCBF1* and *CsCOR* genes in cucumber seedlings under cold stress for 6 h. qRT-PCR was performed simultaneously with three biological replicates and three technical replicates. The value of the water treatment was used as the reference and was set to 1. Error bars denote standard deviations. Different letters indicate significant differences (*p* < 0.05) based on Duncan’s multiple range tests.

A previous study showed that 75 μM IAA enhances the cold tolerance of cucumber seedlings [60]. Here, Figure 4 shows that 75 μM IAA significantly reduced the EL, and accumulation of H_2_O_2_ and O_2_^.−^ caused by cold stress, while 50 μM 1-naphthylphthalamic acid (NPA, a polar transport inhibitor) treatment showed no remarkable difference relative to H_2_O treatment under cold stress (Figure 4A–F). As an auxin response factor, the relative mRNA expression of *CsARF5* was upregulated in IAA-treated seedlings under cold stress. However, no remarkable difference was found in the mRNA expression of *CsARF5* between the NPA and H_2_O treatments (Figure 4G). The mRNA expression levels of *CsCBF1* and *CsCOR* were significantly upregulated in IAA-treated seedlings but downregulated or not influenced in HT-treated seedlings compared with H_2_O-treated seedlings when exposed to cold stress (Figure 4H,I). The latest results are in keeping with the earlier findings [60], so the results further confirm that IAA enhances cold tolerance in cucumber.

### 2.4. CsARF5 Positively Regulates Cold Stress Tolerance of Cucumber

**Figure 5:**
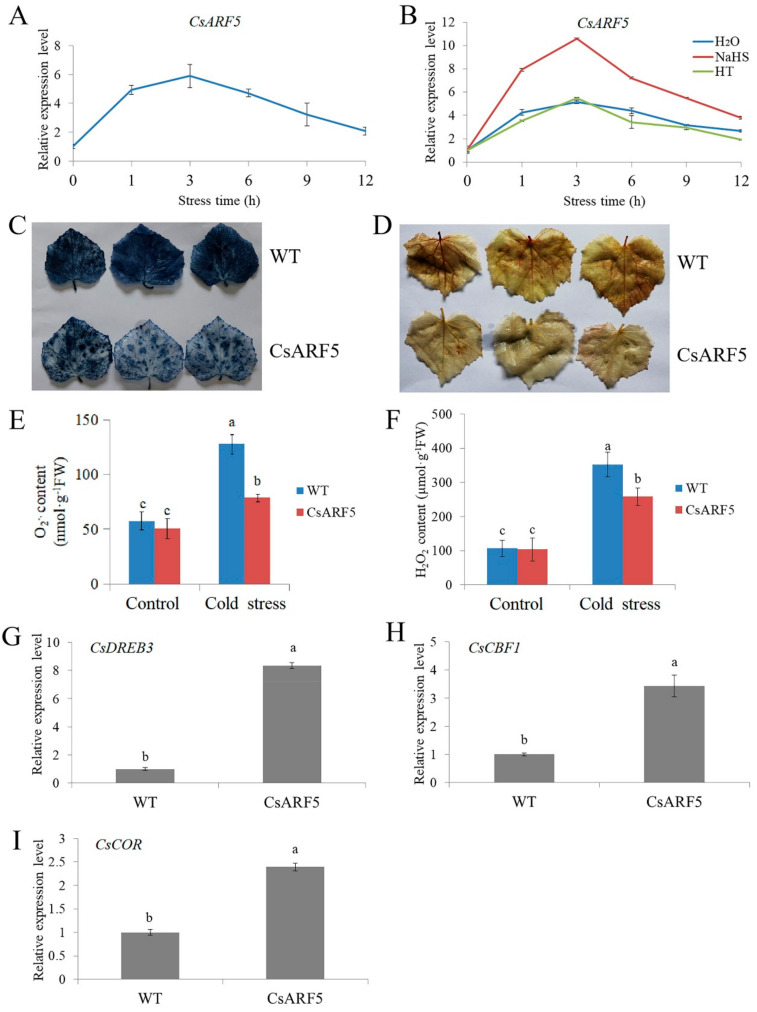
Overexpression of *CsARF5* improves cold tolerance of cucumber. (**A**) Expression of the *CsARF5* gene in cucumber seedlings under cold stress treatment for 12 h. (**B**) Expression of the *CsARF5* gene in cucumber seedlings treated with NaHS or HT under cold stress treatment for 12 h. (**C**,**D**) NBT and DAB staining of empty vector control (WT) and *CsARF5* transient transgenic cucumber leaves treated with cold stress for 12 h. Each genotype contained 5–10 cucumber leaves. The experiments were repeated three times with similar results. A typical picture is shown here. (**E**,**F**) Detection of O_2_^·−^ and H_2_O_2_ contents of transgenic cucumber leaves before (control) and after cold stress treatment for 12 h. (**G**–**I**) Expression of *CsDREB3*, *CsCBF1* and *CsCOR* genes in transgenic cucumber leaves under cold stress for 3 h. qRT-PCR was performed simultaneously with three biological replicates and three technical replicates. The value of WT was used as the reference and was set to 1. Error bars denote standard deviations. Different letters indicate significant differences (*p* < 0.05) based on Duncan’s multiple range tests.

qRT-PCR results showed that cold stress increased the mRNA abundance of the *CsARF5* gene, and the expression reached a peak after seedlings were exposed to cold for 3 h and then decreased gradually (Figure 5A). Compared with the control and HT treatments, NaHS treatment further increased the expression of *CsARF5* (Figure 5B). These results demonstrate that *CsARF5* is responsive to cold stress and H_2_S treatment.

To further explore the role of *CsARF5* in response to cold stress in cucumber, we obtained *CsARF5* transgenic cucumber leaves through *Agrobacterium*-mediated transient genetic transformation (Supplemental Figure S1A). Then, the accumulation of reactive oxygen species (ROS) in transgenic leaves after exposure to cold stress for 12 h were observed, using nitroblue tetrazolium (NBT) and 3, 3-diaminobenzidine (DAB) staining. The results showed that the accumulation of ROS in cucumber leaves overexpressing *CsARF5* (CsARF5) was lower than that of the empty vector control (WT) under cold stress (Figure 5C,D). In addition, the contents of O_2_^·−^ and H_2_O_2_ were measured with biochemical analysis, and the results were in agreement with the NBT and DAB staining images (Figure 5E,F). qRT-PCR results revealed that *CsARF5* overexpression upregulated the mRNA level of cold stress-responsive genes *CsDREB3*, *CsCBF1* and *CsCOR* (Figure 5G–I). These data suggest that *CsARF5* is a positive regulator of cold stress response.

### 2.5. HT Treatment Affects CsARF5-Mediated Cold Stress Tolerance

**Figure 6:**
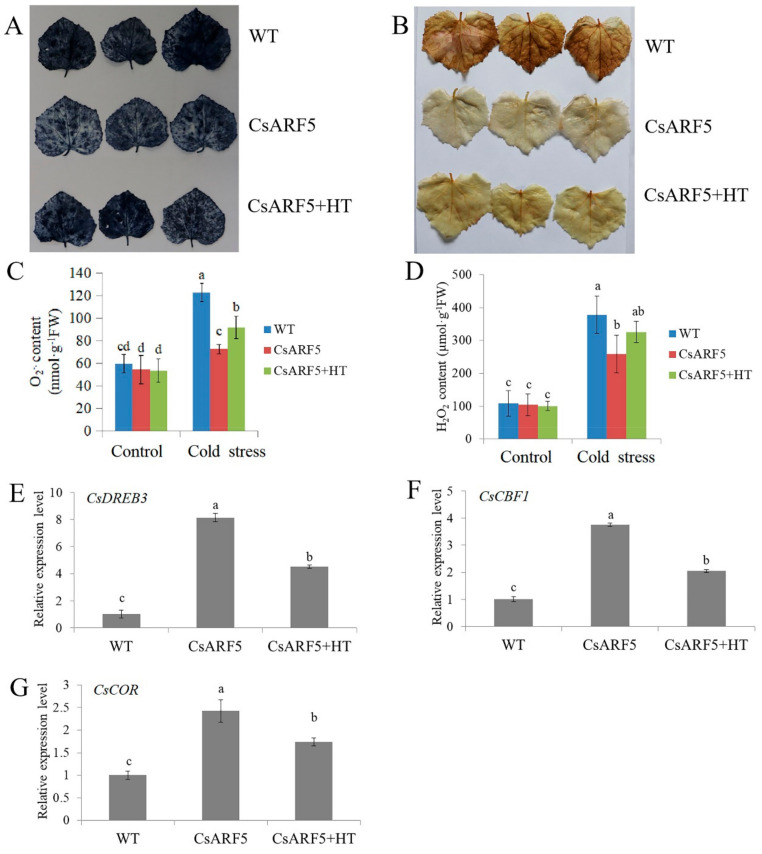
Effect of HT on the cold resistance of *CsARF5* transgenic cucumber. (**A**,**B**) NBT and DAB staining of empty vector control (WT), *CsARF5* transient transgenic cucumber leaves (CsARF5) and CsARF5 sprayed with HT (CsARF5+HT) treated with cold stress for 12 h. Each genotype contained 5–10 cucumber leaves. The experiments were repeated three times with similar results. A typical picture is shown here. (**C**,**D**) Detection of O_2_^·−^ and H_2_O_2_ contents of transgenic cucumber leaves before (control) and after cold stress or HT treatment for 12 h. (**E**–**G**) Expression of *CsDREB3*, *CsCBF1* and *CsCOR* genes in transgenic cucumber leaves under cold stress for 3 h. qRT- PCR was performed simultaneously with three biological replicates and three technical replicates. The value of WT was used as the reference and was set to 1. Error bars denote standard deviations. Different letters indicate significant differences (*p* < 0.05) based on Duncan’s multiple range tests.

Considering that H_2_S induces the expression of *CsARF5*, and that *CsARF5* positively regulates cold stress resistance, the role of *CsARF5* in H_2_S-mediated cold stress was further explored. The H_2_S scavenger HT was applied to *CsARF5*-overexpressing cucumber leaves to observe ROS accumulation. The NBT and DAB staining results showed that the application of HT alleviated the CsARF5-decreased ROS accumulation in detached cucumber leaves (Figure 6A,B). The biochemical analysis for O_2_^.−^ and H_2_O_2_ was consistent with the NBT and DAB staining results (Figure 6C,D). qRT-PCR results showed that the application of HT inhibited the promotion of *CsDREB3*, *CsCBF1* and *CsCOR* mRNA expression levels caused by CsARF5 (Figure 6E–G). These results suggest that the regulation of the cold stress response by *CsARF5* depends on H_2_S. 

### 2.6. Overexpression of CsDREB3 Enhances Cold Stress Tolerance of Cucumber

**Figure 7:**
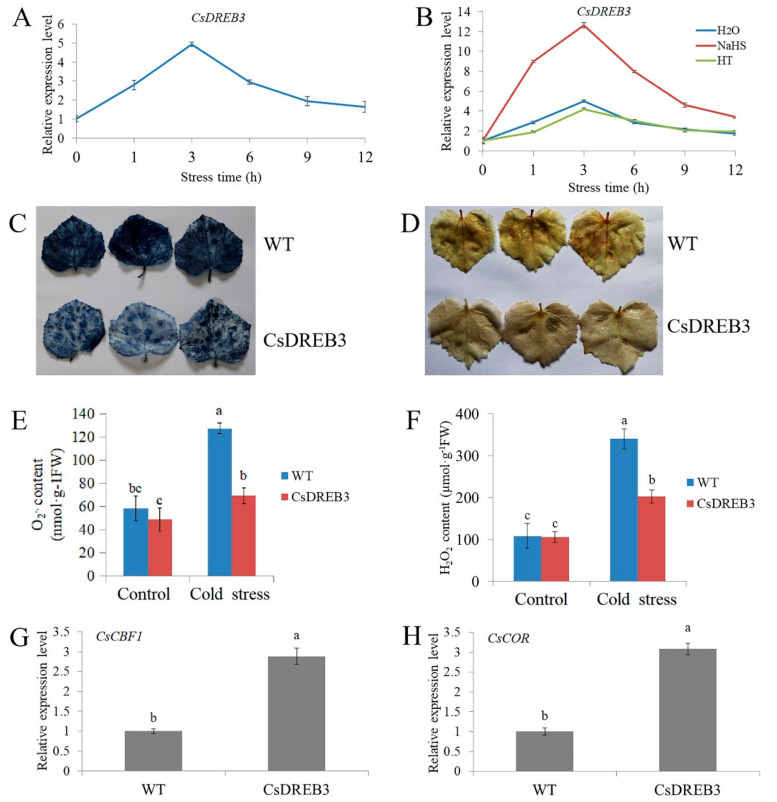
Overexpression of *CsDREB3* improves the cold tolerance of cucumber. (**A**) Expression of the *CsDREB3* gene in cucumber seedlings under cold stress treatment for 12 h. (**B**) Expression of the *CsDREB3* gene in cucumber seedlings treated with NaHS or HT under cold stress treatment for 12 h. (**C**,**D**) NBT and DAB staining of empty vector control (WT) and *CsDREB3* transient transgenic cucumber leaves treated with cold stress for 12 h. Each genotype contained 5–10 cucumber leaves. The experiments were repeated three times with similar results. A typical picture is shown here. (**E**,**F**) Detection of O_2_^·−^ and H_2_O_2_ contents of transgenic cucumber leaves before (control) and after cold stress treatment for 12 h. (**G**,**H**) Expression of *CsCBF1* and *CsCOR* genes in transgenic cucumber leaves under cold stress for 3 h. qRT-PCR was performed simultaneously with three biological replicates and three technical replicates. The value of WT was used as the reference and was set to 1. Error bars denote standard deviations. Different letters indicate significant differences (*p* < 0.05) based on Duncan’s multiple range tests.

DREB/CBF TFs play essential roles in the regulation of the plant cold stress response [61,62]. *CsDREB3* was induced by NaHS (Figure 2B), which prompted us to explore whether CsDREB3 was involved in the cold stress response in cucumber. As shown in Figure 7A, cold stress induced the expression of the *CsDREB3* gene, and the expression reached a peak at 3 h, and then decreased gradually. Compared with the control and HT treatments, NaHS treatment further increased the expression of *CsDREB3* (Figure 7B).

To investigate the role of *CsDREB3* in response to cold stress in cucumber, *CsDREB3* transient transgenic cucumber leaves were obtained (Supplemental Figure S1B). NBT and DAB staining results showed that overexpression of *CsDREB3* decreased ROS accumulation after cold stress treatment (Figure 7C,D). In addition, O_2_^.−^ and H_2_O_2_ detection results also revealed that the accumulation of O_2_^.−^ and H_2_O_2_ was significantly lower in leaves of overexpressing *CsDREB3* than in WT leaves (Figure 7E,F). qRT-PCR results suggested that overexpression of *CsDREB3* increased the expression of *CsCBF1* and *CsCOR* (Figure 7G,H). These data demonstrate that *CsDREB3* positively regulates the cold tolerance of cucumber. 

### 2.7. CsARF5 Directly Activates the Expression of CsDREB3

**Figure 8:**
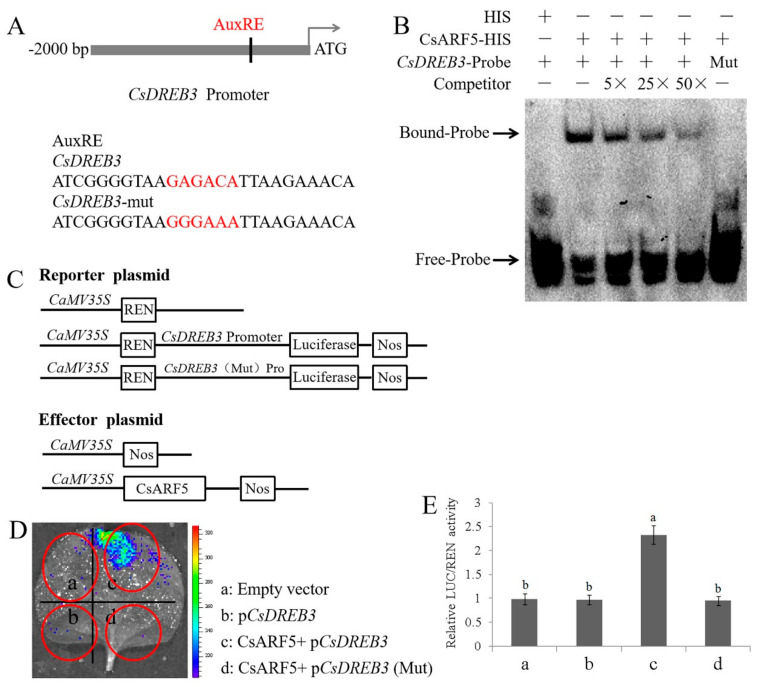
CsARF5 directly modulates the expression of *CsDREB3*. (**A**) Schematic diagram showing the *CsDREB3* promoter probe used for EMSAs. Mutated probe (*CsDREB3*-mut) in which the 5′-GAGACA-3′ motif was replaced by 5′-GGGAAA-3′. (**B**) EMSAs show that CsARF5 binds to the *MdDREB3* promoter. The experiments were repeated three times with similar results. A typical picture is shown here. (**C**) Schematic representation of the LUC reporter vector containing the *CsDREB3* promoter and the effector vectors expressing *CsARF5* under the control of the 35S promoter. (**D**) Dual luciferase tests in tobacco leaves showing that CsARF5 activates *CsDREB3* transcription. Mutated promoter sequence (pCsDREB3-Mut) in which the 5′-GAGACA-3′ motif was replaced by 5′-GGGAAA-3′. The experiments were repeated three times with similar results. A typical picture is shown here. (**E**) LUC/REN activity detection to verify that CsARF5 activates the transcription of *CsDREB3*. Empty vector was used as the reference and set to 1. Error bars denote standard deviations. Different letters indicate significant differences (*p* < 0.05) based on Duncan’s multiple range tests. All experiments were performed three times with similar results, and representative data from one repetition are shown.

CsARF5 acts as an auxin response factor and can bind to the AuxRE motif in the promoters of target genes [27]. Considering the similar expression patterns of *CsARF5* and *CsDREB3* in cold stress and H_2_S treatment, as well as the key role of DREB/CBF TFs in the regulation of the cold stress response, we hypothesized that CsARF5 might be involved in the cold stress response by mediating the expression of *CsDREB3*. Then, the sequence of the *CsDREB3* gene promoter region was analyzed, and a putative AuxRE motif was found (Figure 8A). Fortunately, the direct binding between the CsARF5 protein and the promoter of *CsDREB3* was detected by electromobility shift assay (EMSA) (Figure 8B). To test how CsARF5 regulated the expression of *CsDREB3*, dual luciferase assays in tobacco leaves were performed. The CsARF5 effector construct was expressed under the 35S promoter, and the promoter of *CsDREB3* was fused to the Luc gene as a reporter (Figure 8C). The results showed that co-expression of 35Spro:CsARF5 with CsDREB3pro: Luc led to an obvious increase in luminescence intensity (Figure 8D,E), while the binding site was mutated, and the activation was abolished (Figure 8D,E). These results suggest that CsARF5 trans-activates the expression of *CsDREB3* in cucumber.

## 3. Discussion

H_2_S, as a gaseous signaling molecule, plays a crucial role in plant relevance to various stress conditions, such as low temperature, salt, drought and heavy metals [12,63]. A report showed that the exogenous application of the H_2_S donor NaHS could effectively improve plant growth and stress response [64]. In this study, NaHS treatment reduced the accumulation of ROS and activated the expression of cold stress-responsive genes, thus improving the cold-tolerance of cucumber seedlings (Figure 1), suggesting that the response of H_2_S to cold stress is consistent in different species [64]. Previous reports indicate that H_2_S and phytohormones have synergistic effects on the H_2_S-mediated plant stress response [65]. For example, H_2_S interacts with abscisic acid (ABA) and ethylene and is involved in the plant response to drought stress and in the regulation of stomatal closure [66,67]. Salicylic acid (SA) may play a key role in H_2_S-alleviated heavy metal stress and low-temperature stress [68,69]. Jasmonic acid (JA) stimulation increases the H_2_S level of protective cells and induces stomatal closure in *Vicia faba* [70]. Here, the study found that NaHS treatment promoted IAA synthesis and upregulated the expression of auxin-responsive genes (Figure 2 and Figure 3), indicating that H_2_S may crosstalk with auxin to regulate the cold stress response of cucumber.

Several plant hormones, such as JA, SA and ethylene, have been shown to play key roles in the plant’s response to cold stress [66,67,68,69,71,72,73]. However, the role of auxin under cold stress is limited. Auxin is a crucial phytohormone that is involved in a variety of plant physiological and developmental processes, including the regulation of the cold stress response [74,75]. Previous investigations have determined that cold stress promotes auxin biosynthesis or changes the auxin gradient distribution, thus affecting the root gravity response in *Arabidopsis*, rice and poplar [18,19,76,77]. A recent study showed that the auxin signaling repressor IAA14 plays an important role in integrating microRNAs with auxin and cold reactions in *Arabidopsis* [78]. However, studies on the effects of auxin on plant cold stress response are not sufficient. This study showed that the application of auxin improved cold resistance, whereas the application of the polar transport inhibitor NPA slightly reduced the cold resistance of cucumber seedlings (Figure 4), demonstrating that auxin is a positive regulator of the cold stress response.

As a master regulator of auxin signaling, ARF TFs have been functionally characterized in *Arabidopsis*, rice and *populus trichocarpa* [27,31,32,79]. An increasing number of studies have indicated that ARFs regulate multiple plant developmental processes such as root growth [40,41], flower development [46,47], senescence [48] and stress response [19,80]. Among these processes, the auxin-regulated cold stress response has become a major focus of biotechnology in cucumber, with the task of identifying the cold resistance genes and improving the yield of cucumber. In this study, an ARF TF, CsARF5, was isolated from cucumber, and its expression was induced by NaHS and cold stress treatments, suggesting that CsARF5 may be involved in H_2_S-mediated cold tolerance (Figure 5A, B). 

To investigate the functions of CsARF5, transgenic cucumber leaves overexpressing *CsARF5* were generated (Supplemental Figure S1A). As hypothesized, the physiology and genetic analysis indicated that the overexpression of *CsARF5* decreased the accumulation of ROS and increased the expression level of cold stress-responsive genes, thus improving the cold stress resistance of cucumber (Figure 5). These results suggest that CsARF5 positively regulates the cold stress tolerance of cucumber. Meanwhile, the H_2_S scavenger HT was applied to *CsARF5*-overexpressing cucumber leaves to study the role of *CsARF5* in H_2_S-mediated cold stress. The results showed that HT inhibited the cold stress resistance increased by CsARF5 (Figure 6), indicating that the regulation of the cold stress response by CsARF5 depends on H_2_S.

DREB/CBF proteins play important roles in the regulation of the plant cold stress response [64,65]. In *Arabidopsis*, DREB1A, DREB2C, CBF1, CBF2 and CBF3 are involved in the cold stress response [62,81,82,83]. In rice, OsDREB1A, OsDREB1B, OsDREB1C, OsDREB1F, OsDREB2B and OsDREBL are responsive to cold stress treatment [84,85,86,87]. Here, cucumber *DREB* genes, namely *CsDREB3*, were induced by NaHS and cold stress treatments, and overexpression of *CsDREB3* significantly enhanced the cold stress tolerance of cucumber (Figure 7, Supplemental Figure S1B), revealing that CsDREB3 is a positive regulator of cold stress.

ARF5 is known to function as a transcriptional activator [27]. The remarkable expression of *CsDREB3* in CsARF5 transgenic leaves prompted us to consider whether CsARF5 can directly regulate CsDREB3 expression (Figure 5G). The putative AuxRE elements that were recognized by CsARF5 were searched in the promoter region of *CsDREB3*. Fortunately, one AuxRE site was found, and the results of EMSAs and dual luciferase assays provided evidence to show that CsARF5 could bind to the promoter of *CsDREB3* and activate its expression (Figure 8).

**Figure 9:**
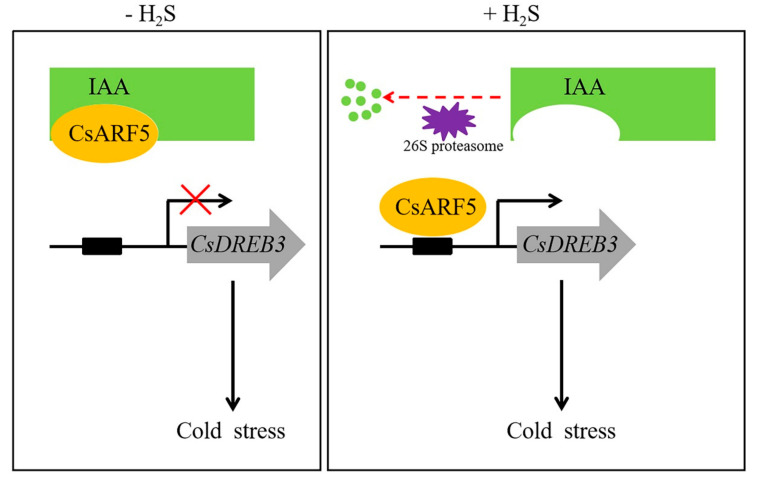
A regulatory model elucidates that H_2_S mediates the cold stress response in cucumber through auxin signaling. In the absence of H_2_S, IAA repressor proteins inhibit the expression of CsARF5, which in turn inhibits the cold stress response mediated by the CsARF5-*CsDREB3* module. In the presence of H_2_S, IAA repressor proteins release CsARF5, which promotes the cold stress response by activating *CsDREB3* expression.

Based on previous studies in model plant species and our results in this study, a working model of CsARF5 regulating the H_2_S-mediated cold stress response was proposed (Figure 9). CsIAA proteins interact with CsARF5 and interfere with the transcriptional regulation of *CsDREB3* by CsARF5. When H_2_S signaling was detected, auxin levels in cucumber were increased, and CsARF5 was freed from the CsIAA protein complex. Then, CsARF5 specifically activated the expression of *CsDREB3* to improve the cold tolerance of cucumber. Our study elucidates the molecular mechanism by which H_2_S regulates the cold stress response in cucumber by mediating auxin signaling, which will provide insights for further studies on the molecular mechanisms by which H_2_S regulates cold stress. A better understanding of the function and signal transduction of CsARF5 in cucumber is helpful to regulate the resistance of cucumber to low-temperature stress to obtain high-quality fruit. 

## 4. Materials and Methods

### 4.1. Plant Material and Growth Conditions

‘Jinyou 35’ cucumber seedlings were used for cold stress treatment and genetic transformation. After soaking and germinating, the cucumber seeds were sown in nutrition bowls and transferred to a climate chamber with a PFD of 600 μmol m^−2^·s^−1^, a 25 °C/16 °C thermo-period, an 11-h photoperiod and 80% relative humidity.

### 4.2. Vector Construction and Transient Transformation

To generate *CsARF5* and *CsDREB3* overexpression vectors, full-length *CsARF5* and *CsDREB3* were inserted into the pCAMBIA1300 plasmid.

For the transient transformation of detached cucumber leaves, cucumber leaves with the same growth conditions were taken, and *Agrobacterium tumefaciens* LBA4404 (Weidi, Shanghai, China) with overexpression vector was injected into cucumber leaves through a medical syringe.

### 4.3. Cold Stress Treatments

To evaluate the effect of H_2_S and IAA on the cold resistance of cucumber, cucumber seedlings with two leaves were foliar sprayed with 1.0 mM NaHS (an H_2_S donor; Shanghai Macklin Biochemical Co., Ltd., Shanghai, China), 0.15 mM HT (a specific scavenger of H_2_S; Sigma-Aldrich, Shanghai, China) and deionized water (H_2_O), respectively, or pretreated with 75 μM IAA (Solarbio, Beijing, China), 50 μM NPA (a polar transport inhibitor of IAA; Shanghai Aladdin Biochemical Technology Co., Ltd., Shanghai, China) or deionized water (H_2_O), respectively. Twenty-four hours later, half of the treatments were exposed to low temperatures (5 °C), and the other half of the seedlings were placed at normal temperatures as the control. The EL and accumulation of ROS were determined at 48 h after exposure of seedlings to 5 °C. Leaf samples of seedlings pretreated with H_2_O and 1.0 mM NaHS were collected from 3 plants (*n* = 3) for transcriptome analysis after 6 h of cold stress.

To compare the difference in cold tolerance between WT and transgenic cucumber leaves, the pCAMBIA1300 empty vectors and overexpressed *CsARF5* and *CsDREB3* vectors were injected into the first leaf, which was just flat of cucumber. Twelve hours later, WT leaves, *CsDREB3* overexpressing leaves, and some *CsARF5* overexpressing leaves were exposed to 5 °C. Other *CsARF5* overexpressing leaves were treated with HT and then exposed to 5 °C after the water droplets on the leaves were absorbed and dried. The gene expression of *CsDREB3*, *CsCBF1* and *CsCOR* in transgenic cucumber leaves was measured at 3 h after exposure to cold stress. NBT staining, DAB staining and ROS content were detected after cold treatment for 12 h.

### 4.4. qRT-PCR Analysis

The transcription levels of *CsARF5*, *CsDREB3*, *CsCBF1* and *CsCOR* were examined using specific primers CsARF5 (qRT)-F/R, CsDREB3 (qRT)-F/R, CsCBF1 (qRT)-F/R and CsCOR (qRT)-F/R, respectively. β-Actin was used as an internal reference. All of the primers used are shown in Supplemental Table S1. qRT-PCR was carried out simultaneously with three biological replicates and three technical replicates.

### 4.5. Detection of EL

EL was detected according to methods described by Dong et al. (2013) [88]. Leaf discs (0.2 g) were immersed in 20 mL deionized water and incubated at 25 °C for 3 h. The electrical conductivity (EC1) was estimated using a conductivity meter (DDB-303A, Shanghai, China). The leaf discs were boiled for 10 min and then cooled to detect EC2. EL was calculated according to the following formula: EL= EC1/EC2 × 100.

### 4.6. IAA Content and FMO Activity Assay

IAA content was measured using high-performance liquid chromatography-triple quadrupole mass spectrometry (Thermo Fisher Scientific, TSQ Quantum Access, San Jose, CA, USA) according to the method of Li et al. (2014) [89], with minor modifications by Zhang et al. (2020) [60]. In brief, leaf samples were extracted with 80% methanol (containing 30 μg·mL^−1^ sodium diethyldithiocarbamate), and the supernatant was retained by rotary evaporation (Shanghai EYELA, N-1210B, Shanghai, China). Pigment and phenolic impurities of samples were removed using trichloromethane and polyvinylpolypyrrolidone (PVPP), respectively. Auxin was further extracted with ethyl acetate, and then the ester phase was collected. Finally, rotation drying at 36 °C and drying were dissolved in 1.0 mL mobile phases (methanol: 0.04% acetic acid = 45:55, *v*/*v*). The filtrate could then be used directly for HPLC–MS analysis. 

The activity of FMO was detected using an enzyme-linked immunosorbent assay (ELISA) kit (Jiangsu Meimian Industrial Co. Ltd., Yancheng, China) as described by Zhang et al. (2020) [60].

### 4.7. Determination of H_2_O_2_ and O_2_^.−^ Contents

H_2_O_2_ content was estimated with the H_2_O_2_ kit (Nanjing Jiancheng Bioengineering Institute, Nanjing, China) according to the instructions. The O_2_^.−^ content was detected according to the method of Wang and Luo (1990) [90]. Cellular H_2_O_2_ and O_2_^.−^ were fluorescently stained with 2′,7′-dichlorodihydrofluorescein diacetate (H_2_DCFDA, the fluorescent probe of H_2_O_2_) (MCE, Cat. No. HY-D0940, Shanghai, China) and dihydroethidium (DHE, O_2_^.−^ fluorescent probe) (Fluorescence Biotechnology Co. Ltd., Cat. No. 15200, Beijing, China), respectively, as described by Galluzzi and Kroemer (2014) [91] and modified by Zhang et al. (2020) [60]. In brief, the samples were infiltrated in a 20 μM H_2_O_2_ fluorescent probe at 25 °C under dark conditions for 30 min. Then the tissues were rinsed with HEPES-NaOH buffer (pH 7.5). Under excitation at 488 nm and emission at 522 nm of an inverted microscope (Leica DMi8), cellular H_2_O_2_ showed obvious green fluorescent coloration. For cellular O_2_^.−^ measurements, the samples were infiltrated in 10 μM DHE at 37 °C under dark conditions for 30 min. After fixation, the tissues were rinsed with Tris-HCl buffer (pH 7.5). O_2_^.−^ showed strong red fluorescence under excitation at 490 nm and emission at 520 nm under an inverted microscope (Leica DMi8).

### 4.8. NBT and DAB Staining

NBT staining of O_2_^.−^ was performed according to the method of Jabs et al. (1996) [92] with minor modifications. The fresh leaves were washed with distilled water, immersed in 0.5 mM NBT in a vacuum and stained at 28 °C for 1 h. Then, the leaves were boiled in ethanol:lactic acid:glycerol (3:1:1) mixed solution to remove pigments, and O_2_^.−^ was visualized in blue-purple coloration. DAB staining of H_2_O_2_ was carried out as described by Thordal-Christensen et al. (1997) [93]. The cleaned fresh leaves were soaked in 1 mM DAB staining solution (pH 3.8) in a vacuum and stained at 28 ℃ for 8 h. Then, the leaves were boiled in ethanol:lactic acid:glycerol (3:1:1) mixed solution to remove pigments and H_2_O_2_ was visualized in reddish-brown coloration.

### 4.9. EMSAs

The CsARF5-HIS fusion protein and biotin labeled probes were prepared for EMSAs. The CsARF5-HIS fusion protein was obtained by inducing *Escherichia coli* BL21 (TransGen Biotech, Beijing, China) with isopropyl β-D-thiogalactoside (IPTG). The biotin-labeled probes were synthesized by Sangon Biotech (Shanghai, China) Co., Ltd. To perform the EMSAs, the fusion protein was mixed with the probe and incubated at 24 °C for 30 min. The protein-probe mixture was separated by nondenatured acrylamide gel electrophoresis.

### 4.10. Dual Luciferase Assay

The promoter sequence of *CsDREB3* was amplified and cloned into pGreenII 0800-LUC to generate the reporter construct pCsDREB3-LUC. The effector plasmid was constructed by inserting full-length *CsARF5* into pGreenII 62-SK. Different plasmid combinations were injected into tobacco (*Nicotiana benthamiana*) leaves by *Agrobacterium tumefaciens* LBA4404. The leaves were sprayed with 100 mM luciferin, and luminescence was detected after being placed in darkness for 3 min. Fluorescence images were obtained with a live imaging system (Xenogen, Alameda, California, USA). The fluorescence activity was determined using a fluorescence activity detection kit (Promega, Madison, WI, USA). 

### 4.11. Statistical Analysis

All experiments were performed at least in triplicate, and the results are expressed as the mean ± standard deviation (SD) of three replicates. The data were analyzed statistically with DPS software. Duncan’s multiple range test was used to compare differences among treatments, and *p* < 0.05 was considered statistically significant.

### 4.12. Accession Numbers

CsARF5 (CsaV3_3G045690), CsDREB3 (CsaV3_2G030880), CsCBF1 (XM_004140746), CsCOR (XM_011659051).

## 5. Conclusions

H_2_S treatment increases cold tolerance and auxin content of cucumber. The auxin response factor CsARF5 directly activates the expression of *CsDREB3* to improve the cold tolerance of cucumber in response to H_2_S treatment. This study elucidates the molecular mechanism by which H_2_S regulates the cold stress response in cucumber by mediating auxin signaling, which will provide insights for further studies on the molecular mechanisms by which H_2_S regulates cold stress.

## Data Availability

The data discussed in this publication have been deposited in NCBI’s Gene Expression Omnibus along with our lab’s previous publication and are accessible through SRA accession: PRJNA579777 (https://www.ncbi.nlm.nih.gov/sra/PRJNA579777, accessed on 26 October 2019).

## Supplementary Materials

The following are available online at https://www.mdpi.com/article/10.3390/ijms222413229/s1.
