# Supplementary material for: Hydrogen Sulfide Improves the Cold Stress Resistance through the CsARF5-CsDREB3 Module in Cucumber
Source: Int J Mol Sci. 2021 Dec 8;22(24):13229. doi: 10.3390/ijms222413229 (PMC8706816; doi:10.3390/ijms222413229)
Supplement: Supplementary file 1 [file ijms-22-13229-s001.zip › ijms-1484878-supplementary.pdf]

Supplemental Tables

Scheme 1. Primers used for gene expression analysis and vector construction.

Primer name	sequence (5’ to 3’)
CsARF5-F	ATGGGCTCTGTGGAAGAGAAG
CsARF5-R	GTATACTCAGGCTGAGGCATG
CsDREB3-F	ATGAGAAATTGGGGGAAATGG
CsDREB3-R	TTTCAACAGTAACACACA
CsCBF1(qRT)-F	TACAGAGGAGTCAGGAGGA
CsCBF1(qRT)-R	AGAATCGGCGAAATTGA
CsCOR(qRT)-F	ACTTTGAGAGGACATTTGATG
CsCOR(qRT)-R	GAAGCTCCAATTTTGACTTG
CsARF5(qRT)-F	GGTGGAAGTACTTTGGTTGATCATG
CsARF5(qRT)-R	AGCAGAGCACAGTTCGTCATAGTTC
CsDREB3(qRT)-F	TGCGCCTCGTGATGTTCAAG
CsDREB3(qRT)-R	ATCCTCCTCTAAGCTCTGCA
β-actin-F	AGAAGATCTGGCATCACA
β-actin-R	TCCAATCCAGACACTGTACT

Supplemental Table 2. The promoter sequence of *CsDREB3*

TAATCTCAATTGAATACAATTATAGGAATTGACATTATATTGTATTTTGTAAAATGAA
AACATGATACTGTAAGAAAGTGAATTAACTATTCAATAGTGATAGAAACAAACAAAT
TTCTATATTTGTCTCTCTATACACAATTCACTATAAAAGACAAATAAAAGAGATAGAT
AAAGATATACATCTAAAGTTATATTTCTTTCTATCTCTGTCTATCTTTATCCATAATTC
ATTATAAAAGACGAATAACAAGGGATAGATAGAGATAATTAACATCTGCAGATAGAC
AGAGATATATGCTTATTTTTGTCTATCTCTATCCACGTTTCTTTATAAAATAATAAAAA
ATAGATGATGATCCTAATCACAATGCAATGGTACAATTAGAATGTTTTATCTGACATA
GATAGATGAAAAGAGATACTTATCTCTACCTATATATGTCCATTATTCACTAATGGTA
AATAATATAACTTTCTGTTAAGATAGGTAAGAGAAAAGTAATAAGTATAAATAAATA
TAATGGTAGTTTAATATAAATGCTATATTTACTATCATGAATGCAAAATTGTATGGTT
TTATTTGATTTAGAGAAAAACATGGTTACGGGACAAATTATTAGTTTTATAAATTGTT
ATAAAATTAATTCTTTGAAATCGTGAACCAATAATAAAGTGTTGCACTTAAACATAGA
ATCTTATGATAGTTATTAGTTTTAAAATATTTAAAATTAATAGATCTAAGATCACATT
AGTTCTAATTAGATTAGAATTAAGTCTTATTTTAGTTTTAATATAACTAAAGATGACA
AATAGGGTGTTCAATCGTAAGGAAACTTTACTAGTAACATTCTGTCTAAGGTTGGGAG
TCCTTAAGTTGAAGATTTACGAAACACCTCCCTACTTAGGAATAAACTTGAAGTCGGA
GTTGAATTGATCTAAGTATTACTCGCATGCAATGTCACTAGGTTTAGTTAAATGGTTT
AATTCACCTAGAAACATTAGTATAGAAACCTTATTACAATAGTTGATAGACATAGACT
TAGATAGATTTATTAGTTGGAATTTAGCCAAGTACACCAAAAACCAAAAACTAATAG
GTAACTTTAGTGGGAAAAGATAAAGGTATATGATAAATCAATTAGCTCTCCCGTCCCT
ATGAGTCCATACTGTCAAATCCATGCTCAACTTCGTGTTGTCTTAGACATGACCTCCC
TTTGGAAAATGTTTGCATGGGTCAATAATAGGGTGAATAGGGGAAGTCGTTCTTAGTA
AGTGAAAGAAGGATGTGTATCAATGTATCCTACGGTCTCCTTCATTCAATCCAACTGT
GAGATTTCTATGGTCCACCTACATGTCATCCTGGAGTGATCATCCCTTCGAGGGTTTG
ACCATATAAATTAGAACACCACAAACTTCAATAACGGAGAGAGTTTCTAGGTTAATA
TTCAACAATTTGCTTTTCTTACGGAAGTCTGTTGAAGCGTACCTTTGGAATCTGAAAA
TGGTAGGGTTAGACTAACGAGATTATTTAGTTAACGAATCCTTAACCAAACAACAGT
AGCTAAGAACTATAAGAATAAAAGTTATTCTAGTATTAATAATTAGATTACTACAAG
AAAATAGGGTTCTTCCAACGCACAAAATTTTATCGGGGTAAGAGACATTAAGAAACA
GGCCTTTCGCCGTTGCCGTAAGTCGTGCATCGAGAATATGAATCTATTCGGATGTATT
GCTTCTGACATCAGACAAAGCTAGACCATCCTTGATGTTGCACACATAAACATCAAA
AAAGGTGAAACAACTAAATTATAATTTCCTTTTCCTCGACATTGTGCAGACAAACGTT
ATGAATGACTTCCATATTTTTGACGTGGACCTTTAAGGAGTTGAGGGAAATGAAATTC
TTATTTAATTGTTTCACCTTTTCTGATGCCATGTGTGAGAACGTCTAGACTATTATGGA
TATTCTTGATGTGTCCACTCACAACGTTAAAGG

Note: Shaded nucleotides indicate the CsARF5 binding site

Supplemental Figures

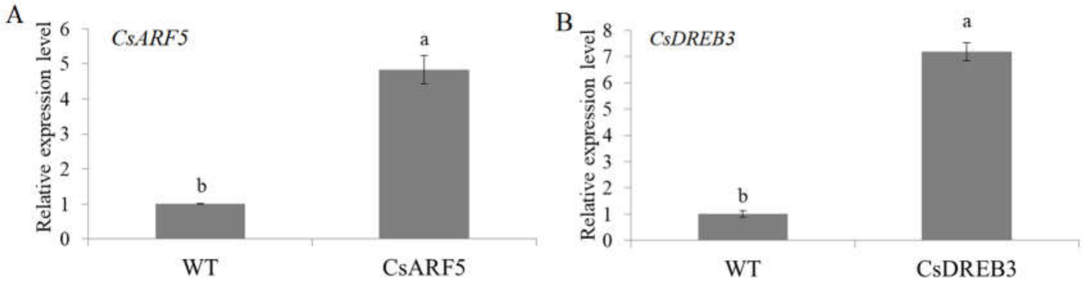

Supplemental Figure 1. Identification of the transient transgenic cucumber leaves.

(A) qRT-PCR analysis the expression of *CsARF5* in transient transgenic cucumber leaves. WT, empty vector control; CsARF5, *CsARF5*-overexpressing cucumber leaves. qRT-PCR was performed in three biological replicates and three technical replicates. The value of WT was used as the reference and was set to 1. Error bars denote standard deviations. Different letters above the bars indicate significant differences ($P < 0.05$) based on Duncan's multiple range tests.

(B) qRT-PCR analysis the expression of *CsDREB3* in transient transgenic cucumber leaves. WT, empty vector control; CsDREB3, *CsDREB3*-overexpressing cucumber leaves. qRT-PCR was performed in three biological replicates and three technical replicates. The value of WT was used as the reference and was set to 1. Error bars denote standard deviations. Different letters above the bars indicate significant differences ($P < 0.05$) based on Duncan's multiple range tests.
